# Can phage–antibiotic combinations overcome uropathogenic *Escherichia coli regrowth*? evidence from in vitro and in vivo models

**DOI:** 10.1186/s12985-026-03067-8

**Published:** 2026-02-06

**Authors:** Salsabil Makky, Assmaa H. Hussein, Amira A. Mohamed, Kareem Essam, Mona M. Agwa, Marwa M. Abd ElAziz, Ayman El-Shibiny

**Affiliations:** 1 Center for Microbiology and Phage Therapy, Zewail City of Science and Technology, Giza, 12578 Egypt; 2 Department of Chemistry of Natural and Microbial Products, Pharmaceutical and Drug Industries Research Institute, National Research Centre, Dokki, 12622 Giza Egypt; 3 Department of Pathology, Faculty of Medicine, Alexandria University, Alexandria, 21131 Egypt; 4 Faculty of Environmental Agricultural Sciences, Arish University, Arish, 45511 Egypt

**Keywords:** Phage therapy, Multidrug-resistant bacteria, ESKAPEE, Uropathogenic *Escherichia coli*, Antibiotic resistance

## Abstract

Phage therapy is currently gaining attention as a promising alternative for treating multi-drug resistant (MDR) bacterial infections, including urinary tract infections (UTIs). However, most studies have reported bacterial regrowth in vitro after hours of co-incubation with phage-host bacteria. In this study, we evaluated whether using a phage alone or combined with gentamicin could delay or prevent bacterial regrowth in vitro, in human urine, and in a rat model. The previously characterized lytic phage vB_Eco_ZCEC08 was combined with gentamicin to target clinical Uropathogenic *Escherichia coli* (UPEC) infection. The minimum inhibitory concentration (MIC) and minimum bactericidal concentration (MBC) of gentamicin against the resistant EC08 clinical isolate were determined, revealing high gentamicin resistance (MIC = MBC, 500 µg/mL). Time-killing assays demonstrated that combining ½ MIC gentamicin (250 µg/mL) with the phage at different multiplicities of infection (MOIs) effectively controlled bacterial growth and prevented regrowth, even after 72 h, in both in vitro culture media and urine. Notably, the phage’s growth exhibited distinct dynamics when used alone versus in combination with gentamicin in both in vitro and in vivo experiments. The combination showed higher replication rates in both urine and the rate model. For the in vivo experiments, bacterial counts showed significant reductions with both phage therapy and combination therapy compared to gentamicin monotherapy. Histopathological analysis of the tissues treated with the combination presented better tissue integrity than either monotherapy. These findings support the potential of phage-antibiotic combinations as an effective strategy against MDR-UPEC infections, highlighting the need for further studies to optimize treatment regimens for clinical applications.

**Graphical abstract:**

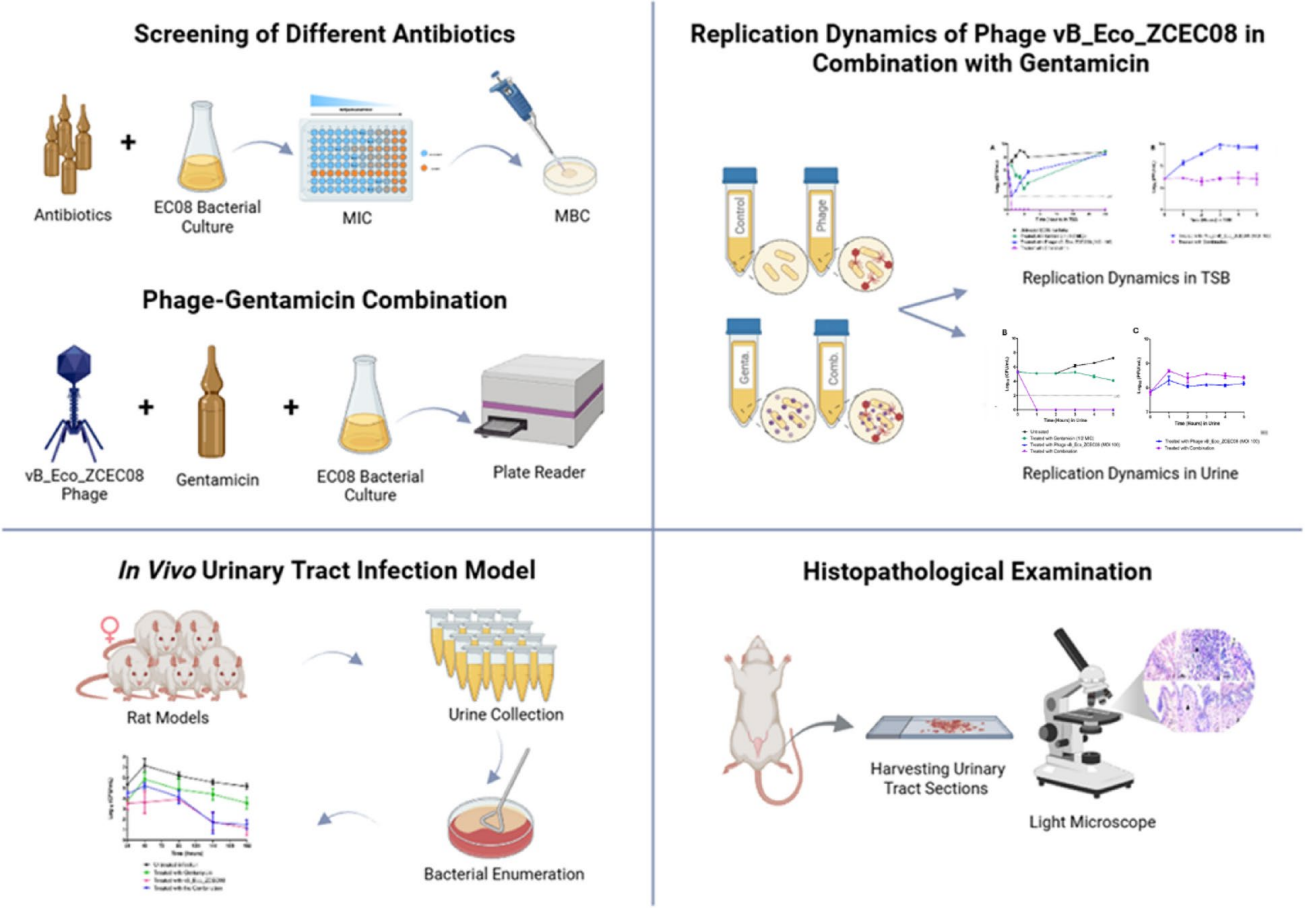

## Introduction

Urinary tract infections (UTIs) are among the most common bacterial infections, accounting for approximately 40% of hospital-acquired infections [12, 25]. The majority of UTIs develop as asymptomatic bacteriuria, which predominantly affects the lower urinary tract. However, bacteria can spread to the bladder and kidneys, increasing the likelihood of upper urinary tract infections, especially in diabetics, the elderly, prepubertal children, pregnant or menopausal women, and patients with vesicoureteral reflux [42]. While UTIs are caused by a variety of pathogens, including fungi, Gram-negative and Gram-positive bacteria, uropathogenic *Escherichia coli* (UPEC) remains the primary pathogen in both uncomplicated and complicated UTIs [14, 42].

UPEC infections are induced via many virulence factors, including toxins, flagella, fimbrial and non-fimbrial adhesins, polysaccharide capsules, surface vesicles, and the iron acquisition system [42]. The spread of resistance genes has recently become a public health concern, especially for members of the *Enterobacteriaceae* family. *E. coli* is the most commonly reported cause of both community and hospital-acquired infections, such as wound infection, neonatal meningitis, sepsis, gastrointestinal tract infection, and most notably UTIs [7, 20]. Increasingly, UPEC shows resistance to numerous antibiotics, leading to long-term hospital stays and excessive treatment costs among UTI patients [30, 42]. This highlights the urgent need for alternative medicine for UTI prevention and treatment rather than relying solely on antibiotics [42].

Virulent bacteriophages (phages) exhibit promising therapeutic capabilities against MDR-UPEC [34]. Since bacteria are genetically mutable, various strategies are employed to bypass bacterial resistance, such as using phage cocktails or phage-antibiotic combinations [40]. For UTI therapeutic preparations, mono-, polyphage cocktails are all reported as good alternatives to ineffective antibiotics [15]. Monophage therapy limits the bacterial targets during treatment procedures, thereby causing low or no side effects on the microbiota; however, it may be associated with antiphage immune responses or the emergence of phage-resistant bacteria in long-term treatments [10]In contrast, two or more virulent phages are used in polyphage therapy to overcome bacterial resistance against each phage as well as the host’s immunogenicity. For instance, Nishikawa et al. [27], used two phages from the *myoviridae* morphotype (T4 and KEP10) to treat UTI in mice after the transurethral induction of UPEC. The findings revealed that approximately 90% of the mice were saved after phage treatment with no remarkable adverse effects. In contrast, the infected but untreated mice died after three days from UTI [27]. Another study reported that a high concentration phage cocktail (10^8^ and 10^7^ PFU/mL) successfully eradicated UPEC infection in a rodent model via the urethral route. However, a lower dose (10^6^ PFU/mL) was insufficient to achieve complete recovery in infected rats [5]. Despite the effectiveness of polyphage cocktail therapy to overcome bacterial resistance and treat UTI, studies indicated the superiority of therapies involving a combination of phages with antibiotics in eradicating biofilm-related infections than those that rely on phage alone [11, 31, 37].

Phage-Antibiotic Synergy (PAS) strategy revealed that sub-lethal doses of certain antibiotics can stimulate bacteria to produce more virulent phages [24]. PAS may also result in unsuccessful bacterial cell divisions, leading to filamentous growth when beta-lactams are present in the environment. This filamentous bacterial growth alters the peptidoglycan layer, thereby increasing sensitivity to phage lysis activity [11, 26]. This would not only affect the bacteria reduction levels but also the bacterial regrowth levels [36]. A research study demonstrated that phage therapy, either alone or in combination with cefotaxime, was highly lethal against both planktonic and biofilm-forming UPEC in vitro, as well as inhibiting biofilm formation on rubber catheters [23]. PAS was also observed to reduce the antibiotic lethal dose from 256 mg/mL to 32 mg/mL when used with 10^7^ PFU/mL of T4 phage [31]. One more form of phage combinations is combining virulent phages with disinfectants such as chlorine compounds or iron antagonists (e.g., Co(II) and Zn(II)) to control biofilm-forming bacteria involved in catheter-associated UTIs [41]. Some UPEC strains were found to depend on iron during biofilm formation, specifically for settling the planktonic bacteria and stabilizing the biofilm polysaccharides; hence, targeting iron acquisition may inhibit biofilm formation (Zalewska-Piątek & Piątek, 2020).

Given the critical importance of controlling phage-resistant bacterial regrowth in advancing phage therapeutics, this study aimed to determine whether the lytic phage vB_Eco_ZCEC08, alone or in combination with gentamicin, could prevent regrowth of MDR-UPEC. To address this, we evaluated phage and phage–antibiotic treatments in vitro (optimized culture media and human urine). In addition, we extended the analysis to an in vivo rat UTI model established via the intraurethral route.

## Materials and methods

### Bacteria and bacteriophage

The bacterial strain and the phage used in both the in vitro and in vivo experiments through this study were previously characterized and published [18]. The strain (EC-08) is an MDR-UPEC isolate with a multiple antibiotic resistance (MAR) index of 0.6. vB_Eco_ZCEC08 is a virulent phage with NCBI ID: PP213477.1.

### Human urine pool collection and Preparation

The urine samples were collected from four healthy volunteers to create a urine pool with a pH of ~ 7. The collection process followed the instructions of the Institutional Review Board (IRB) at Zewail City for Science and Technology. The urine pool was sterilized by filtration using a 0.22 μm syringe filter, warmed at 37 °C, and used freshly. The urine pool was used to assess the stability of vB_Eco_ZCEC08 phage and its replication dynamics with the host bacteria (EC08) in combination with gentamicin over 24 h.

### MIC and MBC assays

Gentamicin antibiotic was tested for its minimum inhibitory concentration (MIC) and minimum bactericidal concentration (MBC) against the EC-08 strain using the standard broth microdilution method following [39], with minor modifications. Briefly, in a 96-well plate, different concentrations of gentamicin (500, 250, 125, 62, 30, 15, 8, 4, 2, and 1 µg/mL) were tested on the bacterial culture with a starting concentration of (5 × 10^5^ CFU/mL). An untreated bacterial culture served as the control, and fresh sterile Tryptone Soy Broth (TSB) served as the blank. The microplate was then statically incubated at 37 °C for 24 h. The static incubation was preferred because it more closely mimics the local physicochemical conditions of the urinary tract [14]. The MIC was determined as the lowest antibiotic concentration that prevented bacterial growth. Regarding the MBC, all wells with no turbidity were observed, and 10 µL of each was spotted on Tryptone Soy Agar (TSA) to enumerate the bacterial count. The plates were then incubated for an additional 24 h at 37 °C. The lowest antibiotic concentration that showed no bacterial growth was reported as the MBC.

### In vitro evaluation of Phage–Gentamicin synergy against UPEC

#### The optimal concentration and MOI for synergy

For a broad screen of phage–gentamicin synergy between phage vB_Eco_ZCEC08 and gentamicin, a time-killing assay was done following [9], with some modifications. The time-killing curves were performed using a FLUOstar Omega microplate reader (BMG LabTech, Germany) to measure bacterial growth levels at an optical density (OD600 nm) with and without treatment, as well as with different doses of antibiotic, phage, and their combination. The screening was performed in three triplicate sets of phage and antibiotic concentrations for 18 h, with OD 600 readings recorded every 2 h. Four groups were designed as follows: group (1), untreated bacterial control, with a starting concentration of (5 × 10^5^ CFU/mL). Group (2) phage control with two different MOIs: high MOI 10 and low MOI 1, independently. Group (3) antibiotic control, in which two concentrations are used: MIC (500 µg/mL) and sub-MIC value (250 µg/mL) of gentamicin, independently. MOI 10 phage and 500 µg/mL gentamicin were chosen because they are the lowest effective concentrations that inhibited bacterial growth based on preliminary experiments. Lastly, group (4) is the combination of antibiotic concentrations with the high and low MOIs. This experiment was conducted in the bacterial growth medium (TSB). MOI 1 and MOI 10 were chosen because they were recommended in the previous study [18].

#### Testing PAS in culture media

The potential synergistic effect of the phage vB_Eco_ZCEC08 with gentamicin was evaluated in culture media by measuring the bacterial and phage count following [9]. For instance, four groups were prepared in TSB media: (1) the bacterial control group (untreated bacteria), (2) the antibiotic control group (bacteria treated with ½ MIC of gentamicin), (3) phage control group (bacteria treated with phage MOI = 100), and (4) a combination group (bacteria treated with ½ MIC of gentamicin and phage MOI = 100). All bacterial cultures used were at the exponential phase (5 × 10^5^ CFU/mL). The experimental groups were incubated at 37 °C for 72 h with frequent agitation (70 rpm). An aliquot was periodically taken to enumerate bacterial and phage counts for each group over a 24-hour period using a 10-fold serial dilution and a spotting assay [17].

#### Testing PAS in urine

The potential synergistic effect of the phage vB_Eco_ZCEC08 with gentamicin was also evaluated in urine by measuring the bacterial and phage count following ; Hay et al., [16]. For instance, the same four groups of the TSB (as described in Sect. "*The optimal concentration and MOI for Synergy"*) were prepared, but using urine instead of TSB culture media. The bacterial cultures used were at the exponential phase (5 × 10^5^ CFU/mL). The experimental groups were incubated at 37 °C for 24 h with frequent agitation (70 rpm), and aliquots were periodically taken to enumerate the bacterial and phage counts from each group over the 24 h using a 10-fold serial dilution and spotting assay [17].

### In vivo urinary tract infection model

#### Experimental animals and UTI induction

The in vivo rat model was conducted according to [22], with some modifications. A total of 25 female albino rats (200–220 gm) were randomly divided into five groups (*n* = 5 per group). Rats were individually acclimated in cages for 48 h, with free access to food and water. Prior to bacterial inoculation, rats were anesthetized with ketamine (65 mg/kg) and xylazine (10 mg/kg), and their bladders were completely evacuated of residual urine. The periurethral region was sterilized with 70% ethanol. Urinary tract infection (UTI) was induced by intravesical instillation of 100 µL containing 1 × 10^7^ CFU/mL of the uropathogenic *E. coli* strain EC-08 through a sterile Teflon catheter (UC-04-TO-W-0; outer diameter 0.4 mm).

#### Treatment regimens and sample collection

The rats were divided into five groups: group 1, standard uninfected controls; group 2, infected untreated controls; group 3, infected and treated with gentamicin (150 mg/kg, intraperitoneally); group 4, infected and treated with 200 µL of 1 × 10^9^ PFU/mL of vB_Eco_ZCEC08 phage (intraperitoneally); and group 5, infected and treated with 200 µL phage (1 × 10^9^ PFU) and gentamicin (150 mg/kg) as combination therapy. All treatments were administered daily until the end of the experiment.

Urine collection from each rat was conducted under sterile conditions using a biosafety cabinet. Each rate was individually transferred into a biosafety cabinet, then handled carefully by a well-trained researcher, and urine was collected in a sterile disposable petri dish by gentle compression of the lower abdominal region [19]. The urine sample was then transferred into a sterile Eppendorf tube using a sterile pipette. The cabinet will be sterilized between animals using 70% ethanol [19].

Urine samples were collected at 24, 48, 96, 144, and 192 h after UTI induction to enumerate bacterial and phage counts. The EC-08 strain was recovered selectively on Eosin Methylene Blue (EMB) agar supplemented with 50 µg/mL ampicillin. After 8 days, all rats were sacrificed. Kidneys and urinary bladders were collected and homogenized for bacterial quantification, and blood samples were collected in heparinized tubes to assess bacterial and phage populations.

#### Histopathological examination

The histopathological examination was done following the protocols in [32]. For instance, at the end of the treatment period, tissue specimens from the urinary tract were collected and fixed in 10% neutral buffered formalin. The fixed tissues were dehydrated in ascending grades of ethanol (70%, 90%, and 100%), cleared in xylene, and embedded in paraffin wax. Serial sections of 4 μm thickness were prepared, dewaxed, and rehydrated through descending grades of ethanol (100%, 90%, and 70%). The sections were stained with hematoxylin and eosin (H&E) according to the manufacturer’s protocol and examined under a light microscope (Olympus CX23).

### Statistical analysis

The experiments were conducted at least in triplicate. The graphs were calculated using GraphPad Prism 5 software. A one-way ANOVA was used to evaluate the significance, with a p-value set at < 0.05.

## Results

### Antibiotic activity against the UPEC model

**Fig. 1:**
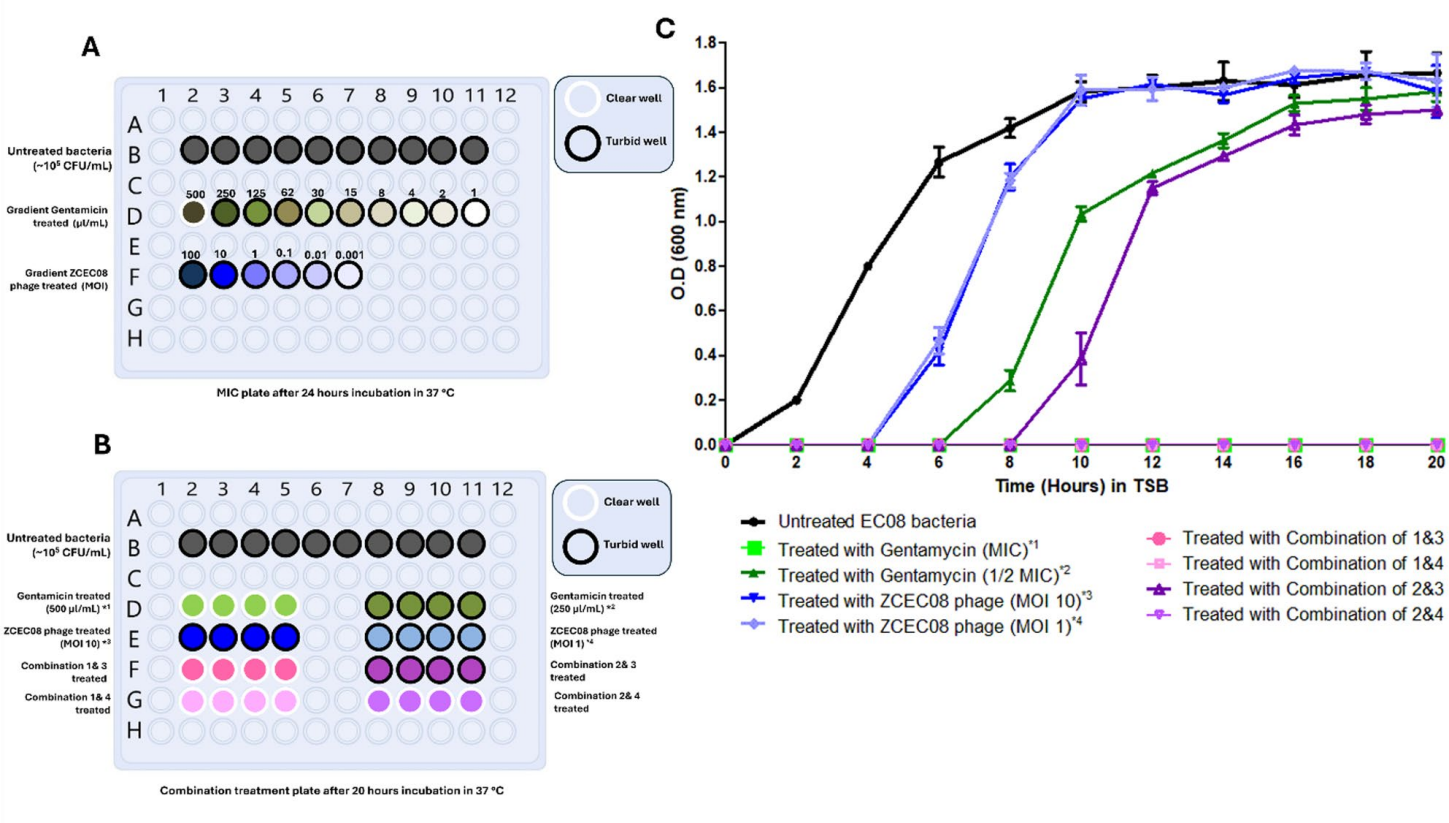
Screening for the combination effect of phage vB_Eco_ZCEC08 with gentamicin. (**A**) The MIC plate after 24 h of incubation at 37 °C, showing the effects of different gentamicin concentrations on ZCEC08 Phage. (**B**) The combination treatment plate after 20 h of incubation at 37 °C. Black-edged wells are turbid, indicating bacterial growth, while white-edged wells are clear, indicating no bacterial growth. (**C**)The graph represents bacterial growth for various treatment groups compared to the untreated control; antibiotic treatment (500 µg/ml and 250 µg/ml gentamicin), bacteria with phage treatment (MOI 10 and 1), combination 1 (MOI 10 of phage and 500 µg/ml gentamicin), combination 2 (MOI 1 of phage and 500 µg/ml gentamicin), combination 3 (MOI 10 of phage and 250 µg/ml gentamicin), and combination 4 (MOI 1 of phage and 250 µg/ml gentamicin)

For the MDR EC-08 isolate, the MIC and MBC of gentamicin were both determined to be 500 µg/mL, as this was the lowest antibiotic concentration that showed no bacterial growth. In contrast, wells treated with gentamicin at concentrations of 250 µg/mL and 125 µg/mL, as well as other lower concentrations, exhibited bacterial growth and turbidity after incubation at 37 °C (Fig. 1A**)**.

### Screening for phage-antibiotic combination

Bacterial growth increased significantly over the experimental period (20 h), reaching an OD600 of ~ 1.8 in the control sample and in samples treated with phage at MOI 10 and MOI 1. Additionally, both the bacteria treated with 250 µL/mL gentamicin and the combination 3 group showed minimal reduction in bacterial growth, reaching an OD_600_ of ~ 1.6. Conversely, the bacteria treated with 500 µL/mL gentamicin, as well as those in combination groups 1, 2, and 4, exhibited no significant bacterial growth over 20 h **(**Fig. 1B& C**)**.

### In vitro evaluation of phage-antibiotic combination

#### At MOI 100 in TSB culture media

**Fig. 2:**
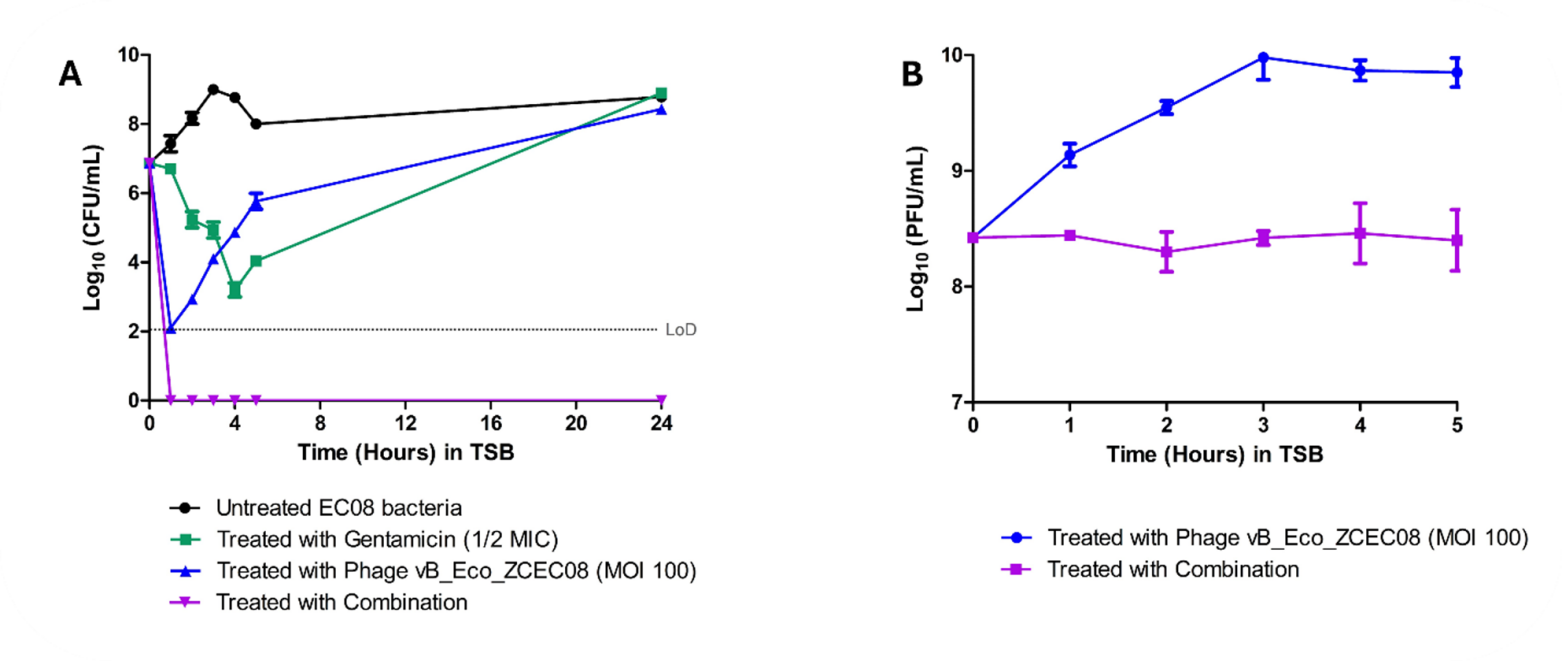
Time-killing curve of phage with gentamicin in TSB at MOI 100. (**A**) Bacterial count after treatment with phage at MOI = 100 (Blue), ½ MIC of gentamicin (Green), and the combination (Violet) in comparison to the control over 24 h. (**B**) Phage count after phage treatment (Blue) and combination treatment (Violet)

For the antibacterial activity of the treatments in culture media, the time-kill curves illustrated the bacterial count over five hours in TSB **(**Fig. 2A**)**. In TSB, both gentamicin alone and the phage–gentamicin combination initially reduced the bacterial count during the early time points (Fig. 2A). However, bacterial regrowth was observed in the gentamicin-treated group after four hours, and in the phage-treated group after only one hour. In contrast, the combination treatment maintained complete suppression of bacterial growth throughout the five-hour experiment, with no detectable regrowth.

For phage growth, the phage titer was higher in the phage-treated group than in the combination group. For instance, the phage-treated group showed a 1.5 log10 increase in phage titer by the end of the experiment. Meanwhile, the bacteria treated with the combination showed minimal to no change in the phage titer **(**Fig. 2B**)**.

#### At MOI 100 in human urine

**Fig. 3:**
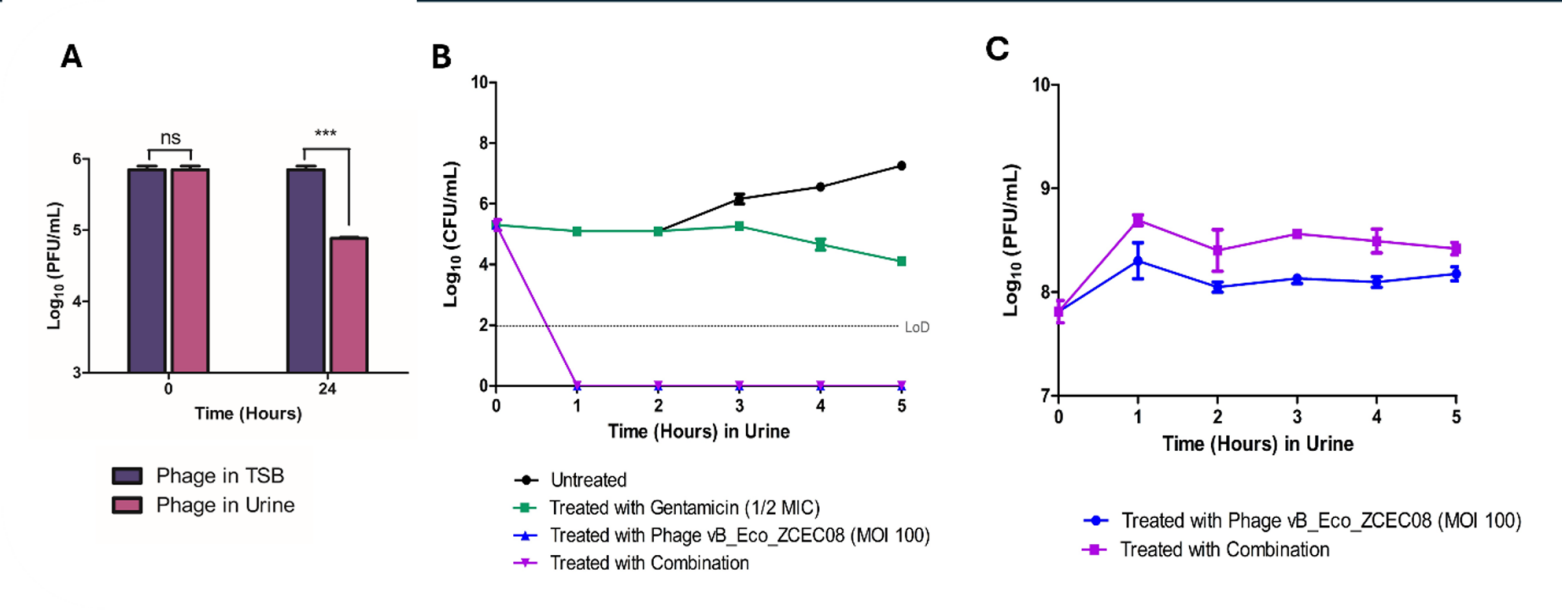
Time-killing curve of phage with gentamicin in Urine. (**A**) Phage stability in Urine. (**B**) Bacterial count after phage treatment at MOI = 100 (Blue), ½ MIC gentamicin (Green), and the combination (Violet) in comparison to the control over 24 h. (**C**) Phage count after phage treatment (Blue), and combination treatment (Violet)

To study the stability of phage vB_Eco_ZCEC08 in urine, the phage was incubated in urine for 24 h. The incubation resulted in a decrease in phage titer from 5 log_10_ to 4 log_10_ PFU/mL, with a reduction of approximately 1 log10 PFU/mL after 24 h **(**Fig. 3A**)**.

For the antibacterial activity of the treatments in urine, the time-killing assays showed that the bacterial count decreased to below the detection limit (LoD) after one hour of incubation in both the phage-treated group and the phage–antibiotic combination group **(**Fig. 3B**)**. In contrast, the bacteria treated with antibiotics alone exhibited an initial decrease over the first five hours to approximately 4 log_10_ CFU/mL, followed by a gradual regrowth over the remainder of the time. Similarly, the bacteria treated with the phage alone exhibited continuous regrowth after the initial reduction, reaching titers comparable to those of untreated controls at approximately 8 log_10_ CFU/mL.

Notably, no regrowth to pre-treatment levels was observed in the phage–antibiotic combination group after the initial decline during the first hour, and complete suppression was maintained until the end of the experiment. Analysis of phage–bacteria dynamics revealed that phage titers were higher in the combination treatment compared to phage treatment alone. In addition, a minimal change in phage titer was observed over the five-hour experiment (Fig. 3C), compared with the same experiment in TSB **(**Fig. 2B**)**.

### In vivo UTI model

#### Microbiological examination

**Fig. 4:**
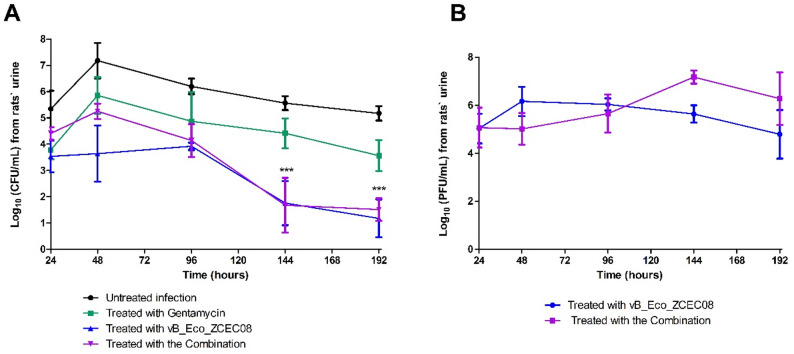
Enumeration of the ZCEC08 phage and UPEC EC–08 strain from collected rat urine samples throughout the in vivo experiment on EMB selective agar medium supplemented with 50 µg/mL ampicillin. (**A**) Bacterial enumeration of the four groups over 192 h: untreated infected rat, rats infected then treated with gentamicin, rats treated with vB_Eco_ZCEC08 phage, and rats treated with a combination of both phage and gentamicin. (**B**) Phage count from the groups treated with the vB_Eco_ZCEC08 phage alone and in the combination treatment. A repeated treatment dose was injected intraperitoneally into the rats after each urine sample collection

For the antibacterial activity of the treatments in vivo, the bacterial count from rats’ urine was tracked over 192 h (six days) in untreated infections and treatments with gentamicin, vB_Eco_ZCEC08 phage, and their combination **(**Fig. 4A**)**. In the untreated group, the bacterial count peaked at approximately 7.1 ± 1.5 Log10 CFU/mL in the first 48 h, followed by a gradual decline, but remained above 5.66 ± 0.6 Log_10_ CFU/mL by 192 h. In the gentamicin treatment group, the bacterial count also increased at the first 48 h but then declined gradually, reaching approximately 4 ± 1.6 Log10 CFU/mL by the end of the experiment. In contrast, the phage treatment displayed a steady decline in bacterial count after 48 h, reaching approximately 1.5 ± 0.96 Log_10_ CFU/mL by 192 h. The combination treatment showed the most pronounced reduction in bacterial count over time compared to the other groups. The phage-antibiotic-treated group showed an initial increase in bacterial count to 5.25 ± 0.65 Log10 CFU/mL at 48 h, which subsequently decreased, reaching approximately 1.7 ± 1.6 Log10 CFU/mL by 192 h.

For the phage-bacteria dynamics alone and in combination with gentamicin, the phage titer was higher in the combination than in phage monotherapy, after 48 h **(**Fig. 4B**)**, which corresponds to the decline in bacterial count **(**Fig. 4A**)**.

#### Histopathological examination

**Fig. 5:**
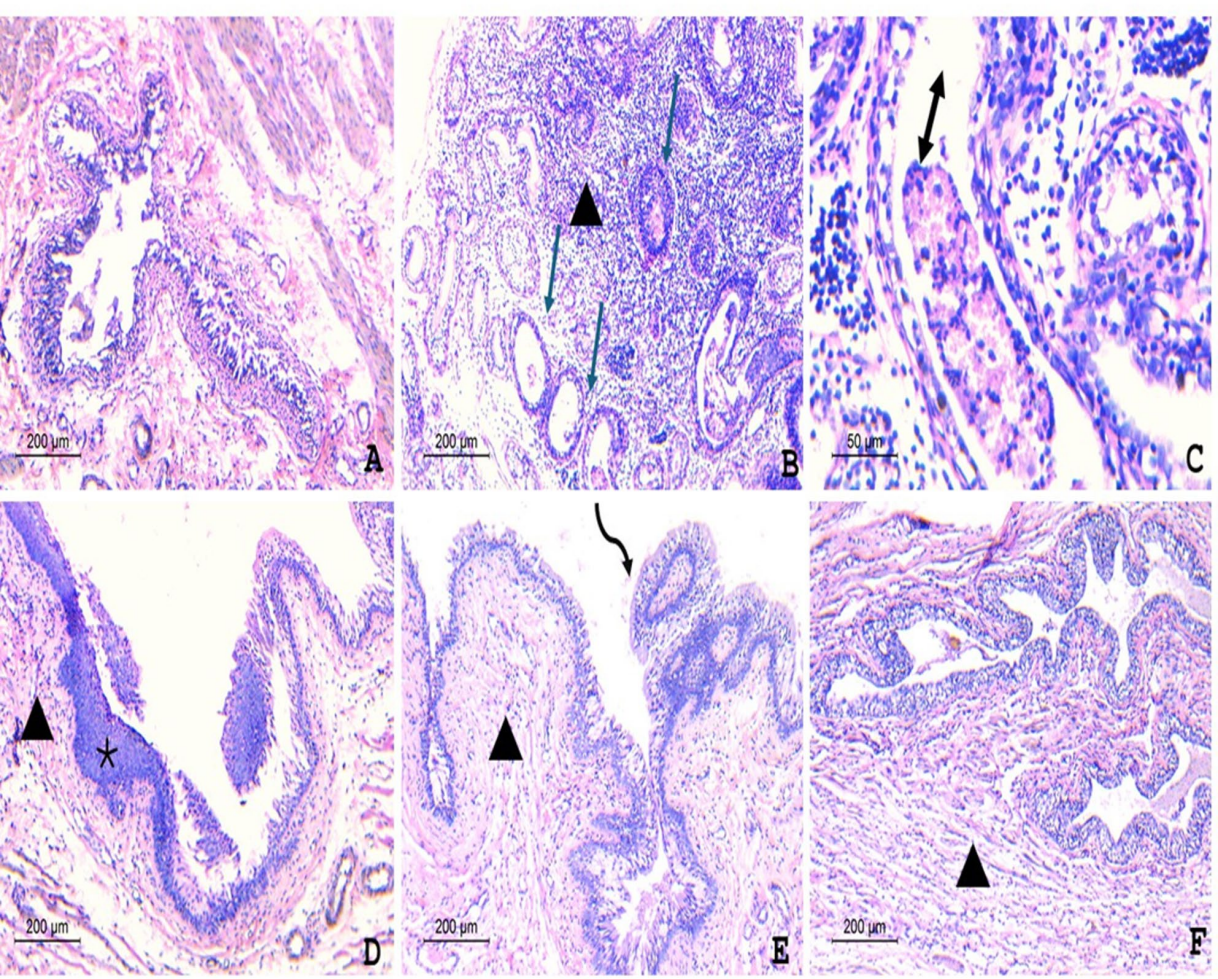
Histopathological examination of urinary bladder tissues (H&E staining). (**A**) Normal control rats (Group 1) show intact urothelium without hyperplasia or inflammation. (**B–C**) Infected untreated rats (Group 2) showing hyperplastic urothelium with cystitis cystica (arrows), dense lymphocytic inflammation (arrowheads), and cystic spaces containing fresh and degenerated neutrophils (double-headed arrows). (**D**) Gentamicin-treated rats (Group 3) show mildly hyperplastic urothelium with foci of squamous metaplasia (asterisk) and mild lymphocytic infiltrate (arrowhead). (**E**) Phage-treated rats (Group 4) show mild urothelial hyperplasia with focal pseudopapillary infoldings (curved arrow) and a mild lymphocytic infiltration (arrowhead). (**F**) Phage–gentamicin co-treated rats (Group 5) show nearly intact urothelium with only mild hyperplasia and mild lymphocytic infiltrate (arrowhead)

Tissue section from group (1) showed a normal urinary bladder with intact urothelium, exhibiting neither hyperplasia nor significant inflammation **(**Fig. 5A**)**. Tissue section from group (2) showed hyperplastic urothelium with the formation of cystitis cystica (arrows) separated by a significant, dense inflammation composed of lymphocytes (arrowhead) **(**Fig. 5B**)**. Higher power illustrated that some of the cystic spaces showed luminal fresh and degenerated neutrophils (double-headed arrow) **(**Fig. 5C**)**. The tissue section from group (3) showed a mildly hyperplastic urothelium with foci of squamous metaplasia (asterisk) without cystitis cystica, with a mild lymphocytic inflammatory infiltrate (arrowhead) **(**Fig. 5D**)**. The tissue section from group (4) showed mild urothelial hyperplasia with focal pseudopapillary infoldings (curved arrow) and a mild lymphocytic infiltrate (arrowhead) **(**Fig. 5E**)**. The tissue section from group (5) showed an intact urothelium with mild hyperplasia and a mild lymphocytic infiltrate **(**Fig. 5F**)**.

## Discussion

Phage therapy has recently gained attention due to the spread of antibiotic-resistant pathogens. Phage cocktails and phage-antibiotic combinations are among the active areas of research, as they overcome the limitations of phage monotherapy. Many studies have reported promising phage-antibiotic combinations in vitro against UPEC. Here, we took a further step by focusing on a single potential phage-antibiotic combination in the UTI rat model. For instance, we studied the potential antibacterial activity of gentamicin and the previously studied *E. coli* phage vB_Eco_ZCEC08 in a UTI rat model. Based on in vitro screening experiments, a sub-MIC concentration of gentamicin (½ MIC) and a high phage input (MOI 100) were selected as working conditions for assessing phage–antibiotic interactions, and their effects were subsequently examined in vivo.

Phage vB_Eco_ZCEC08 was previously studied and showed bacterial regrowth following a short exposure time with the host bacteria [18]. Regrowth was observed across all tested MOIs (0.1, 1, 10, and 100). This makes the phage vB_Eco_ZCEC08 a robust model for studying regrowth when combined with an antibiotic. In the current study, we investigated whether a combination of sub-MIC doses of gentamycin could prevent or delay bacterial regrowth. The question was answered through both in vitro (culture media and human urine) and in vivo (in a rat UTI model) experiments. We found that bacteria treated with the combination showed a reduction in bacterial count and no regrowth to pre-treatment levels, compared with both phage and antibiotic monotherapy, in vitro. The combination also demonstrated controlled bacterial count in vivo, exhibiting better antibacterial activity compared to antibiotic monotherapy, yet similar activity to phage monotherapy. On the other hand, the combination treatment resulted in a more favorable histopathological outcome than both monotherapies, indicating enhanced therapeutic potential.

While TSB is optimal for bacterial growth, body fluids are not. Bacteria multiply at a slower metabolic rate inside the urine [3]. Thus, the experiments were duplicated using TSB and urine. Regarding the stability of phage vB_Eco_ZCEC08 in urine, our results showed a gradual decrease in phage titer with increasing incubation time in human urine. The phage titer decreased by 1 log_10_ after 24 h incubation **(**Fig. 3A**)**. In contrast, phage stability was maintained stable in artificial urine for 48 h in our previous study, with no titer reduction [18]. Another study reported that phages remained stable in urine for at least 12 h [29]. Collectively, these observations suggest that the composition of urine plays a key role in phage stability. While pH may contribute to inactivation, other components of the human urine matrix, such as urea and other metabolites [8], are also likely to influence phage stability.

Growing evidence about phage-antibiotic synergy (PAS) is listed in recent reviews, including those by [13, 33]. For instance, the combination of phage vB_KpnM_P-KP2 and gentamicin was effective in treating *Klebsiella pneumoniae* in a lung infection in vitro and in vivo rat model, when compared to the monotherapy [38]. Additionally, the combination of phage phiPT1 and gentamicin was more effective in treating Salmonella enteritidis infections in vitro than monotherapy [2]. PAS was also achieved when the same phage was combined with other antibiotics, including aztreonam, cefixime, and ciprofloxacin, but no effect was reported with meropenem and colistin [2].

The result of the current study confirms previous reports that PAS can produce synergistic effects and limit bacterial regrowth. In our in vitro assays, the combination of phage and gentamicin was effective in suppressing bacterial growth for the first five hours of infection in both optimized culture medium and urine **(**Figs. 2A and 3B**)**. Notably, no regrowth to pre-treatment levels was observed after 72 h of co-incubation in both conditions. Several mechanisms may explain the synergy between phages and gentamicin. As an aminoglycoside, gentamicin inhibits protein synthesis by binding to the 30 S ribosomal subunit, thereby impairing bacterial growth and increasing susceptibility to phage infection. Sublethal concentrations of gentamicin have been reported to affect phage replication by slowing host metabolism, thereby prolonging the bacterial lysis cycle and allowing higher phage yields [35]. Reduced phage titers in combination treatments have been previously reported and are attributed to antibiotic-induced reductions in bacterial host density. This might lead to limited phage replication despite overall enhanced antibacterial efficacy [1]. This was also observed in our in vitro urine and in vivo rat model data **(**Figs. 3C and 4B**)**. The higher phage growth in the rat model is likely due to the presence of bacteria that phage can still replicate within. In particular, in vivo conditions and complex biological fluids such as urine may provide metabolic and ecological contexts that enhance phage–antibiotic synergy (Oechslin et al., [28]; Pereira et al., [29].

In contrast to the PAS observed in vitro, the combination therapy and phage monotherapy exhibited similar trends in controlling bacterial growth in the rat UTI model **(**Fig. 4A**)**. This indicates that phage alone, particularly at a high MOI, was sufficient to achieve robust control of infection and was more effective, thereby masking any additive benefit of gentamicin. In vivo phage therapy studies commonly employ high phage doses (e.g., MOIs of 100 or greater) to overcome physiological constraints such as diffusion barriers and immune clearance [28]. Another possibility is the relatively low dose of gentamicin used. The gentamicin dose could have been suboptimal for achieving antibacterial synergy in vivo. Future studies should investigate higher or optimized dosing regimens of the combination treatment in vivo to overcome this limitation and more effectively evaluate potential synergistic effects. Moreover, high-throughput screening methods or deep learning models could be used to predict the optimal treatment doses [21].

Although there was no significant difference in the bacterial count between the phage monotherapy and the combination therapy in vivo, a marked difference was observed at the histopathological level. Bladder tissues from the combination group showed an intact urothelium with only mild hyperplasia and a minimal lymphocytic infiltrate, indicating near-complete mucosal recovery [4]. In contrast, the phage monotherapy group exhibited mild urothelial hyperplasia with pseudopapillary infoldings and a mild lymphocytic infiltrate, indicating ongoing tissue remodeling [6]. This dissociation between bacterial burden and tissue integrity implies that the treatment used in the combination group not only controlled bacterial growth but also facilitated the resolution of inflammation and promoted epithelial regeneration.

Overall, our study highlights the importance of evaluating phage–antibiotic combinations in physiologically relevant environments, such as urine and animal models, rather than relying solely on optimized culture media conditions. Since urinary tract infections naturally occur within a complex urine matrix, assessing phage stability and therapeutic efficacy in this context provides more clinically meaningful insights. One limitation of the in vivo model is the use of a single high phage MOI and a single gentamicin dose. Also, the high phage MOI may have masked the antibiotic’s additive or synergistic effects on the bacteria. These results support the potential of PAS as an alternative strategy for combating MDR-UPEC infections, highlighting the need for further optimization of dosing regimens and validation in clinically relevant models.

## Conclusion

Our study evaluated the efficacy of combining the lytic phage vB_Eco_ZCEC08 with gentamicin against uropathogenic *E. coli in vitro* (in culture media and human urine) and in vivo (in a rat urinary tract infection model), aiming to explore alternative strategies for combating MDR-UPEC infections. In vitro, both monotherapies initially controlled bacterial growth but were subsequently followed by regrowth, whereas the combination prevented regrowth and achieved a greater reduction in bacterial growth. In vivo, the phage monotherapy and combination outperformed gentamicin monotherapy in bacterial clearance. Additionally, the combination produced more favorable histopathological outcomes than monotherapy, indicating superior therapeutic potential. The combination also enhanced the phage dynamics in vivo. These findings support the rationale for phage–antibiotic combinations as a promising approach for treating MDR infections and controlling secondary bacterial growth. Further studies are needed to optimize dosing, frequency, and delivery strategies, enabling preclinical validation and eventual clinical translation.

## Data Availability

The datasets analyzed during this study are available from the corresponding author upon reasonable request.
